# Redefining Clinical Skills in History Taking in Association With Epidemiological Assessment of Risk Factors, and Diagnosis of Patients With Cardiovascular Diseases With a Special Emphasis on COVID-19

**DOI:** 10.7759/cureus.30829

**Published:** 2022-10-29

**Authors:** Akshaya Narasimman, Sonali G Choudhari

**Affiliations:** 1 Department of Community Medicine, Jawaharlal Nehru Medical College, Datta Meghe Institute of Medical Sciences, Wardha, IND; 2 School of Epidemiology and Public Health, Department of Community Medicine, Jawaharlal Nehru Medical College, Datta Meghe Institute of Medical Sciences, Wardha, IND

**Keywords:** covid and cardiovascular complications, doctor – patient, clinical skills education, cardiovascular diseases, history taking

## Abstract

Cardiovascular diseases were the leading cause of death in the world prior to the COVID-19 pandemic. A wide range of risk factors may precipitate a cardiovascular disease and therefore multiple aspects of the patient's history may lend a hand in the diagnosis of the specific stage of cardiovascular disease that is presented by the patient. This article will give a general review of the knowledge and skillsets needed by a clinician to distinguish and at the same time correlate the different presenting symptoms and the potential cardiac issue associated with them. History taking is a very essential and critical clinical skill that is of paramount assistance in diagnosing and treating the patient with the right management therapies to find a cure for the disease. Basic approaches in the evaluation of the physical condition and cardiac assessment are important skills in healthcare that help decrease mortality in everyday life and therefore are needed to be learned efficiently. Lifestyle changes and modern standard of living especially in a developing country like India contribute majorly to the evolution of this disease in the population as well as the eating habits and addictions which play a vital role in the progression of the disease. The aim of this article is also to give an outline of various risk factors and recent etiological agents by analyzing the epidemiological variation and patterns that can be ruled out or considered associated with the cardiovascular related-symptomatology and relevant history of the patient to confirm a diagnosis by investigations which will direct the clinician towards specific treatments and recovery of the patient. A special topic of understanding would be the COVID-19-associated cardiovascular complications which have been recently discovered and studied as a result of the pandemic.

## Introduction and background

Concerns and the quest to know about cardiovascular diseases (CVDs), which have been the main cause of mortality in industrialized nations, and the spread of the epidemic now to emerging countries have been seen from different perspectives by different researchers. The exact pathophysiology that is common to all is atheromatous vascular disease, which leads to peripheral vascular disease, cerebrovascular disease, coronary artery disease (CAD), and ultimately heart failure and cardiac arrhythmias. Long-term studies have identified several major risk factors for these disorders, including lack of physical exercise, regular tobacco consumption, metabolic disorders like diabetes mellitus, high blood pressure, central abdominal obesity, psychosocial factors, irregular timings of unhealthy meals, excessive alcohol consumption, and high levels of low-density lipoprotein or even hypercholesterolemia [1]. A patient's overall health, the existence of venous distention or pulsation, as well as the quality and rate of their arterial pulses, the look of their mucous membranes, and auscultation of their heart rate and rhythm, are all used to evaluate their cardiovascular system [2].

Unmodifiable risk factors are not changeable and include, heredity or genetic makeup, diabetes insipidus, and age. The deterioration of the body with age becomes a predisposing factor for the majority of chronic illnesses. The body is subjected to a variety of stresses as we age, including free radicals produced by the body, which promote the degeneration of organs and cell functioning. People who have a family history of coagulopathies and heart disease are more likely to acquire CVDs, according to epidemiological studies. Additionally, a person with type 1 (juvenile) diabetes experiences impairments in several bodily processes, most notably tolerance to glucose and metabolism of fats. The person is more prone to acquire CVDs as a result of such metabolic disorders. The increased prevalence of cardiovascular disease and stroke has also been attributed to risk factors such as severe migraines, abrupt anxiety, and hormonal contraceptive usage [3-5].

When compared to inactivity, increased physical activity lowers the chance of having cardiovascular disease. Only people who are completely inactive or who have pre-existing health issues are at risk for acute cardiovascular events with abrupt, vigorous-intensity physical exercise, albeit there may be a threshold at which activity levels transmit higher risk. These dangers might be reduced by introducing the new activity and having a doctor check you out before starting an exercise program. Therefore, a personal history of physical fitness, duration, and intensity of exercise is as important as a history of a stationary lifestyle. It may also be influenced by the type of occupation of the person, i.e., desk-bound jobs, manually stressful jobs, etc [6].

Diabetes mellitus has a lot of complications on the heart as increased oxidative stress, impaired protein kinase C signaling, and a rise in advanced glycation end-products cause inflammation and constriction of blood vessels, thrombus formation, and vascular dysfunction. These conditions are brought on by increased blood glucose levels, resistance to insulin, and an excess of fatty acids [7].

An increase in cholesterol causes atherosclerosis of blood vessels and hypertension causes tearing of blood vessels due to excessive force of circulation and pumping of the heart triggered by different external and internal factors. Therefore, detailed history in consideration of all these will help in reaching the right diagnosis and treatment for the patient [8,9].

Clinical skills are acquired by assessing the risk factors, observing the signs presented and the history given by the patient in addition to reviewing the clinical investigations and coming to a provisional diagnosis, which may later be discussed among peers for a differential opinion, and finally coming to the most probable diagnosis. Accuracy and precision of the probable diagnosis need a wide range of knowledge of the various trends in the epidemiological distribution of the manifestations of cardiovascular diseases among different sections of the communities. These may be correctly deduced with the information provided by surveys and registered data about the disease prevalence and may be managed with different levels of treatment procedures and therapy.

## Review

A clinician's approach to history taking and management

Rapport Building with the Patients: A Perspective

Communication is the new age key to success and a very important part of the doctor-patient relationship. A good history comes with great communicative skills, established with the right amount of empathy, sympathy, and understanding the clinician builds with the patient. The main aim of the medical professional is to make the patient comfortable by creating the best environment possible so that there is trust built between them. The most effective way to extract the maximum and relevant information regarding the problems and the history is by communication in the vernacular language. This makes the patient feel the interest of the doctor, an association of being connected by the mother tongue which makes the patient talk about his/her problems transparently. Asking about the type of profession has also been proven to be effective by some researchers [10,11].

Airway, Breathing, Circulation, Disability, and Exposure (ABCDE) Approach to Emergency Management

**Figure 1:**
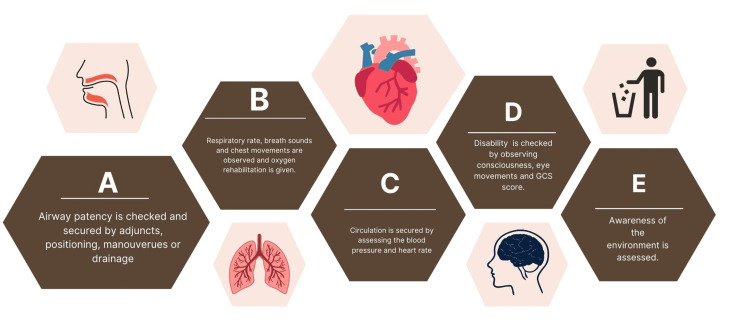
ABCDE Approach for Stabilising a Patient in an Emergency GCS- Glasgow Coma Scale Image credit: Author Akshaya Narasimman with reference from Thim et al. [13] (Open access)

This approach is very crucial along with the efficient assessment of the heart and history of the patient. It provides for the provision of a life-saving intervention and the division of challenging clinical problems into simpler parts. It creates a shared situational awareness among all healthcare professionals and allows time to determine a definitive diagnosis and course of action [12]. ABCDE's approach for immediate yet effective management of the patient is shown in Figure 1. An effective method for restoration of basic life support is a fundamental skill set required in a clinician and thus with or without equipment, help must be given by following such a basic approach in cases of extreme distress or crisis.

The approach to recalling the eight elements of medical problems to ask the patient for obtaining a history of the presenting illness is shown in Table 1. This may provide a structured way of asking for important information about the clinical condition shown by the patient. Clinical aspects of the elaboration of symptoms for accurate diagnosis are remembered by the popularly used mnemonics by clinicians given in Table 2. 

**Table 1:** A sequential and comprehensive format of history taking in cardiovascular diseases Source: Tagney and Younker [14] (Open access)

FORMAT OF TAKING A DETAILED HISTORY OF A PATIENT
Demographic information and reference number: Name, age, gender, address, occupation, socioeconomic status, religion, and caste (to rule out specific diseases), unique hospital reference number for identification, and contact details. Exceptions: In mentally compromised, unconscious, sometimes children, elders, and speech-deficient patients, the nearest relative of the patient is asked for the patient’s history.
Chief complaints: In cardiac tissues, a patient usually complains of chest pain, breathlessness, light-headedness/dizziness, cold sweat, heart palpitations, swollen feet, cyanosis, overall body weakness, and fatigue.
History of presenting illness: This is a detailed explanation of the chief complaints to find the pattern of the presentations and to know the precise cause of these symptoms.
Past history: This includes past admissions in hospitals and major or minor surgeries undergone by the patient. Vaccination and physical fitness regimes may also be a part of this. History of infection with COVID-19 is also included in light of the pandemic.
Family history: History of heart diseases, any other congenital anomalies, or the presence of genetically transmitted diseases for making a pedigree chart. Also, the life expectancy of relatives and family members gives an idea about the livelihood of the patient.
Personal history: Educational, employment status, bowel habits, dietary habits, and any interests that may be medically relevant.
Social history: Addictions like alcohol consumption, smoking, mental status of happiness, or depression due to the influence of socializing factors.
General examination: Assessment of temperature, pulse, respiratory rate, blood pressure, and pallor, icterus, cyanosis, clubbing, lymphadenopathy, and edema (PICCLEE).
Differential diagnosis and management: Some heart diseases can be concluded with this detailed history taking, which has been discussed in the following sections.

**Table 2:** Mnemonics widely followed in asking patients about their symptoms Source: Tagney and Younker [14] (Open access)

“PQRSTU” MNEMONIC	“OLD CART” MNEMONIC
P- Palliative means when the symptoms get better or worse.	O- Onset of the symptoms gradually or suddenly.
Q- Quantity/quality of the type of symptoms that is felt by the patient and if it affects the day-to-day activities of the patient.	L- Location on the body.
R- Radiating/region means which region of the body is affected and does it radiate to other parts of the body.	D- Duration of the persistence of the symptoms.
S- Severity on a scale of 1 to 10, with 10 being the most severe.	C- Characteristics of severity, quantity, and quality of the symptoms.
T- Timing or onset of the problems.	A- Aggravating factors refer to the factors due to which the symptoms are enhanced and associated factors.
U- Understanding what the patient understands by the problem.	R- Relieving factors are the things that cause relaxation of symptoms.
	T- Treatment has been taken by the patient previously or any remedy tried.

There is great relevance of the conversation-making skills of the clinician for good rapport building with the patient. It is also important for ethical decisions that the doctor needs to take after the diagnosis or while diagnosing the condition regarding the course of the treatment that needs to be taken while easing the patient throughout the process. Direct and indirect ways of asking diagnostically relevant questions to form a precise judgment and differential diagnosis based on the sharing level of the patient is an important communication skill that should be learned [15,16].

Symptomatology and Differential Diagnosis in a Cardiac Patient: An Outlook

Acute coronary syndrome (ACS) is identified in 10% of individuals with acute chest discomfort. Many hospitals stay among low-risk patients might be avoided with the early, precise prediction of the probability of ACS in these individuals using the clinical examination, whilst high-risk patients could be treated right away [17-19].

Dyspnea is the awareness of the shortness of breath and may be due to several respiratory and cardiac reasons. Orthopnea and paroxysmal nocturnal dyspnea are indications of left ventricular failure which occurs as breathlessness during the night while lying flat on the back [20,21].

Another important symptom is palpitations, which could be rapid or stationary. Dyspnea and palpitations both are associated with anxiety, physical exertion, and emotions [22,23].

Understanding the myocardial compromise brought on by myocardial necrosis, myocardial stunning, and mechanical consequences such as heart muscle bursting, ventricular septal defect, and ventricular free wall fissures are among the factors that contribute to the pathogenesis of heart failure development at the time of sudden myocardial infarction and admission to the hospital [24,25].

One percent of newborns are born with congenital heart disease (CHD), a type of birth abnormality. Although CHD can be induced by environmental exposures to teratogenic effects, the finding of a high-frequency risk and hereditary variants of the illness, and even the well-described link of CHD with chromosomal aberrations, strongly imply a genomic substrate for the condition [26,27].

Pregnant females who have indications of heart disease from routine hospital visits are also susceptible to suffering most commonly from cardiomyopathies and heart failures. The hormonal imbalances lead to overcompensation by the circulatory system with the same amount of blood in the body. This overloads the functioning of the heart leading to its failure or hypertrophy as a defense mechanism [28,29].

Psychiatric analysis and counseling should be recommended by doctors for patients with terminal or severe heart conditions, especially old people, who would need medical assistance in coping with grave situations like serious heart disease [30]. 

Cardiovascular Diseases as a Sequel of COVID-19

The most common symptoms and signs that a medical clinician should be keenly observant of in suspecting a cardiac condition are tachycardia, palpitations, chest pain, dyspnea on exertion, and intolerance to physical exercise. Studies, experiments, and case reports have led to a very basic understanding of the association between the virus and the cardiovascular system. 

In the COVID-19 era, acute myocarditis can show a wide range of clinical severity and provides considerable diagnostic difficulty. Chest discomfort, breathlessness, irregular heart rhythm, and acute left ventricular failure can all occur in COVID-19 patients [31-33]. The ECG irregularities due to inflammation of the myocardium and findings of PR segment and ST segment depressions and elevations, non-specific ST segment-T wave abnormalities, T wave inversions, and troponin level peaks are difficult to diagnose in the background of COVID-19 raised markers, and often lead to bad prognosis and results. Acute myocardial infarction is known to be associated closely with Covid-19 diagnosed patients due to the rupture of atheromatous plaques in the blood vessels surrounding the heart leading to severe myocardial infarction. Hospital-acquired pneumonia due to bacterial and viral causes like Influenza has been recorded to affect the COVID-19-affected population which is hospitalized, very commonly. Thrombophilia and extreme inflammatory conditions lead to high susceptibility to myocardial infarctions [34,35].

The most obvious sign of COVID-19 infection might be acute cardiac failure. According to one study, less percentage of individuals who initially appear with COVID-19 may have acute heart failure than cardiomyopathy. With or without a history of hypertension or any cardiological dysfunction, this condition is seen to precipitate [36]. 

Patients with COVID-19 infections have reported experiencing a wide variety of dysrhythmias. Such individuals typically have sinus tachycardia, which has several underlying factors such as feverishness, decreased perfusion, low oxygen delivery to the heart, and anxiety. According to one research, 44 percent of COVID-19 ICU patients and 17 percent of hospitalized patients both had dysrhythmias. Viral infection can lead to dysrhythmias because of hypoxia, inflammatory stress, and aberrant metabolism. In the differential diagnosis, the physician should take damage to the myocardium and acute myocarditis into account if dysrhythmias are linked to an increase in blood troponin. Over seven percent of individuals with COVID-19 may also present with palpitations in such conditions [37-39].

Heart problems are the initial clinical sign of COVID-19 in some people who don't have normal symptoms like cough or fever. During COVID-19, myocardial damage is independently associated with an increased death rate. Furthermore, a condition resembling Kawasaki illness has been observed in children who may have COVID-19 [40-42].

People diagnosed with COVID-19 having concomitant cardiovascular disorders are at risk for significant drug-disease interactions. We can better understand the possible processes behind COVID-19 by fusing our understanding of the mutating biological and structural compositions and characteristics of the virus and the host-virus interactions along with clinical results and we open the door to the accurate diagnosis and creation of prophylactic and therapeutic measures [43].

With such viral complications affecting the functioning of the heart, the symptomatology and the signs perceived by the clinician may or may not be directed toward the exact cause of the disease, leading to misdiagnosis and mortality, especially in economically growing countries.

## Conclusions

In-depth knowledge of the manifestations of the numerous cardiovascular conditions and extensive observatory skills to look for the disease even with atypical presentations is the need of the hour and a significant area for learning. A proper sequence and protocol of standard clinical procedure compiled in this article may be of assistance in the hands-on practice of medicine where different patients showcase common or uncommon signs which need to be investigated thoroughly to narrow down the precipitating factor of causation of the disease. Awareness of the significance of various blood components, their irregular elevation and reduction, monitoring of electrocardiograms, and isolating of applicable etiology from the history of the patient is important and makes the process challenging with a wide window of inaccuracies. Being the leading cause of systemic death globally, cardiovascular diseases need to be screened, diagnosed, and treated with precision to be accountable for the pressing burden on the medical fraternity, clinicians, and medical assistants to this day. Diagnosis of a cardiovascular disease not only requires the appropriate clinical skillsets but also the knowledge of current epidemiological trends in the spread of diseases, its incidence in different population groups, and the ability to deduce the causative factor in order to treat the patients in the best possible way.
